# An immunohistochemical perspective of PPAR*β* and one of its putative targets PDK1 in normal ovaries, benign and malignant ovarian tumours

**DOI:** 10.1038/sj.bjc.6604306

**Published:** 2008-03-18

**Authors:** N Ahmed, C Riley, M A Quinn

**Affiliations:** 1 Women's Cancer Research Centre, Royal Women's Hospital, Melbourne, Victoria, Australia; 2 Department of Obstetrics and Gynaecology, University of Melbourne, Melbourne, Victoria, Australia; 3 Department of Surgery, University of Melbourne, Melbourne, Victoria, Australia; 4 Victorian Adult Burns Unit, Alfred Hospital, Melbourne, Victoria, Australia

**Keywords:** ovarian carcinoma, peroxisome proliferating-activated receptor, phosphoinositide-dependent protein kinase 1, metastasis, differentiation

## Abstract

Peroxisome proliferator-activated receptor *β* (PPAR*β*) is a member of the nuclear hormone receptor family and is a ligand-activated transcription factor with few known molecular targets including 3-phosphoinositide-dependent protein kinase 1(PDK1). In view of the association of PPAR*β* and PDK1 with cancer, we have examined the expression of PPAR*β* and PDK1 in normal ovaries and different histological grades of ovarian tumours. Normal ovaries, benign, borderline, grades 1, 2 and 3 ovarian tumours of serous, muciuous, endometrioid, clear cell and mixed subtypes were analysed by immunohistochemistry for PPAR*β* and PDK1 expression. All normal ovarian tissues, benign, borderline and grade 1 tumours showed PPAR*β* staining localised in the epithelium and stroma. Staining was predominantly nuclear, but some degree of cytoplasmic staining was also evident. Approximately 20% of grades 2 and 3 tumours lacked PPAR*β* staining, whereas the rest displayed some degree of nuclear and cytoplasmic staining of the scattered epithelium and stroma. The extent of epithelial and stromal PPAR*β* staining was significantly different among the normal and the histological grades of tumours (*χ*^2^=59.25, d.f.=25, *P*<0.001; *χ*^2^=64.48, d.f.=25, *P*<0.001). Significantly different staining of PPAR*β* was observed in the epithelium and stroma of benign and borderline tumours compared with grades 1, 2 and 3 tumours (*χ*^2^=11.28, d.f.=4, *P*<0.05; *χ*^2^=16.15, d.f.=4, *P*<0.005). In contrast, PDK1 immunostaining was absent in 9 out of 10 normal ovaries. Weak staining for PDK1 was observed in one normal ovary and 40% of benign ovarian tumours. All borderline and malignant ovarian tumours showed positive cytoplasmic and membrane PDK1 staining. Staining of PDK1 was confined to the epithelium and the blood vessels, and no apparent staining of the stroma was evident. Significantly different PDK1 staining was observed between the benign/borderline and malignant ovarian tumours (*χ*^2^=22.45, d.f.=5, *P*<0.001). In some borderline and high-grade tumours, staining of the reactive stroma was also evident. Our results suggest that unlike the colon, the endometrial, head and neck carcinomas, overexpression of PPAR*β* does not occur in ovarian tumours. However, overexpression of PDK1 was evident in borderline and low- to high-grade ovarian tumours and is consistent with its known role in tumorigenesis.

Peroxisome proliferator-activated receptors (PPARs) are members of the nuclear receptor superfamily (Feige *et al*, 2006). The three closely related PPAR isoforms identified as PPAR*α* (NR1C1), PPAR*β* (NR1C2) and PPAR*γ* (NR1C3) are encoded by separate genes and fulfil specific functions (Michalik *et al*, 2003). As transcription factors requiring activation, the PPARs modulate the expression of target genes in the cytoplasm or in the nucleus in response to ligand binding. Physiological ligands of PPARs include fatty acids and their derivatives, leukotrienes and prostaglandins. DNA binding by PPAR requires heterodimerisation with the retinoid X receptor (RXR), the receptor for 9-*cis* retinoic acid. The PPAR-RXR heterodimer binds to the promoter region of its target genes on a specific DNA sequence element, termed the peroxisome proliferators-responsive element (Nahle, 2004), and upon ligand-dependent activation stimulates the transcription of genes by recruiting co-activators.

Peroxisome proliferator-activated receptor *β* is ubiquitously expressed and has been implicated in adipose tissue formation (Bastie *et al*, 2000), brain development (Peters *et al*, 2000), placental function (Barak *et al*, 2002), wound healing (Di-Poi *et al*, 2003) and atherosclerosis (Planavila *et al*, 2005). The antiapoptotic role of PPAR*β* contributes to efficient wound healing in the skin and is mediated by the transcriptional upregulation of integrin-linked kinase (ILK) and PDK1 (Di-Poi *et al*, 2002). The role of PPAR*β* has been controversial in the field of cancer. In colon cancer, PPAR*β* expression was shown to be associated with intestinal tumorigenesis, with increased mRNA levels being observed in several colorectal cancer cell lines and colon tumours (Park *et al*, 2001a; Burdick *et al*, 2006). A PPAR*β*-deficient colon cancer cell line was defective in establishing tumours when grown as xenografts in nude mice (Park *et al*, 2001a), and the activation of PPAR*β* by a synthetic ligand in mice pre-disposed to intestinal tumorigenesis (Apc^Min^ mice) resulted in a significant increase in the number and size of intestinal polyps (Gupta *et al*, 2004). Heightened expression of PPAR*β* has also been demonstrated in head and neck carcinomas (Jaeckel *et al*, 2001) and endometrial carcinomas (Tong *et al*, 2000). Peroxisome proliferator-activated receptor *β* agonist has been shown to act as a tumour promoter in a mammary carcinogenesis model (Yin *et al*, 2005) and to proliferate the growth of human hepatocarcinoma cell line HepG2 (Glinghammar *et al*, 2003). Contrary to these reports, however, the presence of PPAR*β* expression has also been shown to decrease during colon carcinogenesis in both the Min mutant and chemically induced mouse models, where colon polyp formation was significantly greater in mice null for PPAR*β* expression (Harman *et al*, 2004). Consistent with that, others have recently demonstrated APC^Min^ PPAR*β* null mice to have an increased predisposition to intestinal tumorigenesis (Reed *et al*, 2004). In the same way, the ligand activation of PPAR*β* in PPAR*β*^+/+^ mice resulted in increased expression of colonocyte differentiation and apoptosis, inhibition of colon polyp multiplicity, effects not observed in PPAR*β*^−/−^ mice (Marin *et al*, 2006). In addition, PPAR*β*-dependent regulation of ubiquitin has been shown to attenuate skin carcinogenesis (Kim *et al*, 2004). These reports suggest the growth inhibitory and differentiation role of PPAR*β* in colon and skin carcinogenesis and contradict the growth-promoting reports described previously.

The function of activated PPAR*β* is dependent on the activities of its putative downstream targets PDK1 and ILK, both of which act as oncogenes when expressed in mammary epithelial cells (Somasiri *et al*, 2001; Zeng *et al*, 2002), and are involved in the activation of cell proliferation and survival pathways. Peroxisome proliferator-activated receptor *β*-mediated activation of ILK and PDK1 is controlled at the transcriptional level (Di-Poi *et al*, 2002, 2005; Tan *et al*, 2003) and is closely connected to the decrease of PTEN expression (Han *et al*, 2005), commonly lost in tumours, including those of ovarian origin (Dinulescu *et al*, 2005). This activation cascade triggers the Akt1 survival pathway normally seen in the activation of growth factor receptors or integrin-linked signals (Ahmed *et al*, 2005). We have recently demonstrated increased ILK expression in high-grade ovarian tumours and epithelial ovarian cancer cell lines (Ahmed *et al*, 2003). Moreover, Akt, the most extensively studied downstream target of both ILK and PDK1, has also been shown to be overexpressed in ovarian carcinomas (Arboleda *et al*, 2003). Recently, it has been demonstrated that targeting PDK1 by antisense oligonucleotides blocks the proliferation of U-87 glioblastoma cells by promoting apoptosis (Flynn *et al*, 2000b). Consistent with that, the expression of PDK1 in mouse mammary epithelial cells has been shown to drive neoplastic transformation through the activation of Akt1 and PKC*α* pathways (Zeng *et al*, 2002). In addition, the expression of PDK1 in mammary cancer cells was shown to modulate MMP-2 activation with concomitant modulation of ECM proteins decorin and collagen (Xie *et al*, 2006). These data suggest that abnormal expression and activation of PDK1 initiate neoplastic transformation and provide a framework for the cells towards a tumorigenic phenotype.

In addition to PPAR*β* activation, the aberrant activation of growth factor receptors and their downstream target such as activation of PI-3 kinase (Roymans and Slegers, 2001) also controls PDK1 (Toker and Newton, 2000; Vanhaesebroeck and Alessi, 2000; Fresno Vara *et al*, 2004). Many cancers including ovarian cancer, possess elevated levels of PI-3 kinase (Miled *et al*, 2007). Cells having increased PI-3 kinase activity possess mutated PTEN and have elevated levels of PKB (Alessi *et al*, 1997) and PDK1 (Bayascas *et al*, 2005) activity, which in turn activates several protein serine/threonine kinases, including PKC (Dutil *et al*, 1998; Le Good *et al*, 1998), ribosomal S6 kinase (Alessi *et al*, 1998), SGK (Pullen *et al*, 1998), Rho kinase (Flynn *et al*, 2000a) and PAK1 (King *et al*, 2000), all of which are associated with increased invasion and metastasis (Park *et al*, 2001b). Recently, it has been demonstrated that reducing the expression of PKB in PTEN-deficient cells reduces aggressive growth and promotes apoptosis (Stiles *et al*, 2002), whereas reducing the expression of PDK1 in heterozygous PTEN^+/−^ mice markedly protects these animals from developing a wide range of tumours (Bayascas *et al*, 2005). These results suggest PDK1 as a key mediator of neoplasia and validate PDK1 as a promising anticancer target for the prevention of tumour formation.

In this study, we have examined the expression of PPAR*β* and PDK1 in normal ovaries, benign tumours and the histological grades of ovarian tumours. We demonstrate that nuclear and cytoplasmic PPAR*β* is located in the epithelial and stromal cells of normal ovaries, benign tumours and low- to high-grade ovarian carcinomas. On the other hand, normal ovaries and a bulk of benign ovarian tumours demonstrate no significant expression of PDK1, but enhanced cytoplasmic and membrane expression of PDK1 was observed in borderline and low- to high-grade ovarian tumours. Our results suggest that PPAR*β* may have a distinct role in normal and malignant ovarian physiology, whereas PDK1 may be associated with ovarian tumour progression and metastasis. To our knowledge, this is the first study that describes a detailed expression profile of PPAR*β* and PDK1 in normal ovaries, benign tumours and all histological grades of ovarian carcinomas.

## MATERIALS AND METHODS

### Antibodies and reagents

Rabbit polyclonal antibodies against PPAR*β* and PDK1 were obtained from Santa Cruz Biotechnology Inc. (sc-7197; Santa Cruz, CA, USA) and Cell Signaling Technology (3062; Brisbane, QLD, Australia).

### Tissues

This study was approved by the Research and Human Ethics Committee (HEC no. 02/30) of The Royal Women's Hospital, Melbourne, Australia. Ovarian cancer patients with serous, mucinous, endometrioid, clear cell carcinoma and mixed subtypes were included in the study. The histopathological diagnosis and tumour grades were determined by two staff pathologists as part of clinical diagnosis. Histological grading of ovarian carcinoma was determined by the method described previously (Silverberg, 2000). Normal ovaries were removed from patients undergoing surgery as a result of suspicious ultrasound images, palpable abdominal masses and/or family history after the provision of a participant information statement and with informed consent. The histopathological analysis of normal ovaries was evaluated by the staff pathologists in the hospital.

Archival tissues were obtained from the Department of Pathology, Royal Women's Hospital, from women who presented for surgery after the provision of a participant information statement and with informed consent. With few exceptions, the majority of the cases (∼79%) evaluated for the immunohistochemical expression of PPAR*β* and PDK1 were the same (8 out of 10 in normal, 9 out of 10 in benign, 8 out of 9 in grade 1 and 8 out of 11 and 8 out of 11 in grades 2 and 3). A small difference in the sampling (∼21%) among normal and different pathological grades of cancer was made where there was an inadequate amount of specimens needed to complete both studies. This was done with the purpose of having an adequate number of samples for statistical analysis.

### Description of patients included in the PPAR*β* study

The mean age of healthy volunteers participating in the PPAR*β* study was 51 years, whereas that of women presenting with benign and borderline tumours was 61 years. The mean age of women with cancer was 60 years. Out of the 10 benign tumours, 8 were of the serous subtype, whereas 2 were of the mucinous type. Eight out of 10 borderline tumours were in the serous group, whereas 2 were of the mucinous subtype. Seven out of nine grade 1 malignancies used for the PPAR*β* study were of endometrioid subtype, one was mucinous and one of mixed subtype (endometrioid, mucinous, serous). Seven of these patients had Stage 1 disease, whereas one had Stage 2 and the other had Stage 3. Among grade 2 tumours, eight had serous ovarian carcinoma, whereas two were of mixed subtype (endometrioid, mucinous, clear cell carcinoma). All of these patients except one (Stage 1) were of Stage 3. Eight out of 10 grade 3 tumours were of serous subtype, whereas one was endometrioid and the other of clear cell subtype. Seven of these grade 3 patients were Stage 3, one was Stage 2 and the remaining two were Stage 4.

### Description of women included in the PDK1 study

The mean age of the control group participating in the PDK1 study was 51 years, whereas that of women presenting with benign and borderline tumours was 70 years. The mean age of women with cancer was 57 years. Eight out of 10 benign and borderline tumours in each group were of the serous subtype, whereas two were of the mucinous type. Six out of nine grade 1 tumours were of endometrioid subtype, two were mucinous and one was of the mixed subtype (endometrioid, mucinous, serous). Seven of these patients had Stage 1 disease, whereas two were Stage 2. Among grade 2 tumours, five were serous, two were mucinous, one transitional and three of mixed subtype (endometrioid, mucinous, clear cell carcinoma). Seven of these tumours were Stage 3 and only four were Stage 1. Seven out of 11 grade 3 cancer patients were of serous subtype, whereas two were endometrioid and the other two of clear cell subtype. Seven of these grade 3 tumours were Stage 3, one was Stage 2 and the remaining three were Stage 4.

### Immunohistochemistry

Immunohistochemical analysis on ovarian tissues was performed as described previously (Ahmed *et al*, 2002a, 2002b). Briefly, paraffin sections were cut at 4 *μ*m thickness, mounted on silane-coated slides and incubated overnight at 37°C. Sections were washed with water after two changes of xylene and three changes of ethanol. Antigen retrieval was performed using citrate buffer (pH 6.0) and sections were held in Tris-buffered saline (TBS). Endogenous peroxidase activity was removed using 3% hydrogen peroxide in methanol. The sections were incubated for 1 h in primary antibody diluted 1:200 in 1% BSA in Tris buffer (100 mM, pH 7.6). Antibody binding was amplified using biotin and streptavidin HRP (Chemicon, Temecula, CA, USA) for 15 min each and the complex visualised using diaminobenzidine (DAB). Nuclei were lightly stained with Mayer's haematoxylin. Non-immune rabbit serum was used as a control. The specificity of PPAR*β* and PDK1 antibodies was evaluated by Western blot in ovarian tumour homogenates and ovarian cancer cell lysates. In both cases, right molecular weight bands were observed.

Sections were assessed microscopically for positive DAB staining. Two observers (NA and CR) independently evaluated the immunostaining results, and the degree of staining was scored in a blind manner. The concordance ratio was >95% in each case. Differences in opinion were resolved by re-evaluating the sections and, in some cases, by reaching a consensus with the assistance of a third evaluator. Four sections were assessed per tissue, and the cellular distribution of staining was determined.

## INTERPRETATION OF STAINING RESULTS

Immunohistochemistry results were evaluated by two independent observers using the following parameters:

1. Staining pattern, that is, localisation of immunoreactive PPAR*β* and PDK1 in the cytoplasm, membrane and/or nucleus of tumour epithelial and stromal cells;
2. Presence and extent of staining using the following scale: for each specimen, the positive staining extent was scored in 5 grades, namely 0 (⩽10%), 1 (⩾11–25%), 2 (⩾26–50%), 3 (⩾51–75%), 4 (⩾76–90%) and 5 (⩾ 90–100%). The extent of staining was further classified into three grades: negative (0), low (1, 2), moderate (3) and high (4, 5).

### Statistical analysis

The significance of the extent of immunohistochemical staining between normal, benign, borderline and histological grades 1, 2 and 3 ovarian tumours was determined by the non-parametric *χ*^2^ contingency test. *P*<0.05 was considered statistically significant.

## RESULTS

### Immunohistochemical expression of PPAR*β* in ovarian tissues

#### Normal ovaries

**Figure 1:**
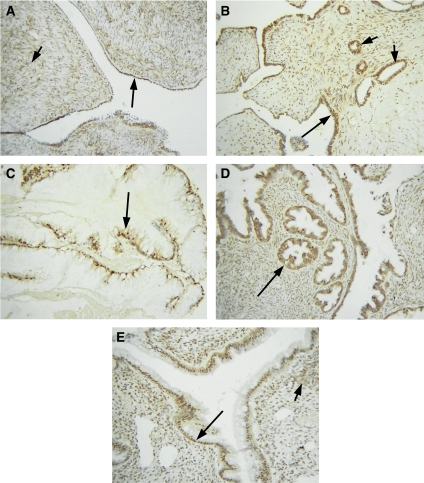
Expression of PPAR*β* in normal ovaries, benign and borderline tumours. Archival ovarian tissues were stained by the immunoperoxidase method as discussed in the Materials and methods section. (**A**) Normal ovary, long arrow showing continuous expression of PPAR*β* in the epithelium, whereas the short arrow illustrates PPAR*β* expression in the stroma. (**B**) Benign serous ovarian tumour, long arrow displays nuclear epithelial staining while short arrows indicate epithelial staining of the inclusion cysts; (**C**) benign mucinous tumour, arrow indicates nuclear epithelial staining. (**D**) Borderline, serous and (**E**) borderline mucinuous tumours. Long arrows in each case indicate positive epithelial PPAR*β* staining, whereas short arrow illustrates positive staining of the stroma.

All normal ovarian sections examined showed staining of PPAR*β* on the surface epithelium (Figure 1A). In most of the cases (7 out of 10), 50% of the epithelial nuclei were positively stained. Extensive nuclear staining of the cortical stroma was also evident. In some sections, contents of the inclusion cysts and fallopian tubes also stained positively. Positive staining of the macrophages was also evident in some sections.

#### Benign and borderline tumours

Benign tumours exhibited extensive epithelial staining with 6 out of 10 tumours exhibiting >50% of nuclear epithelial staining (Table 1, Figure 1B). The associated stromal tissues also showed some degree of staining confined to both nucleus and cytoplasm. Epithelial staining of the inclusion cysts was also evident in some sections (Figure 1B). The pattern of staining in borderline ovarian tumours was similar to benign tumours exhibiting >50% of epithelial staining. Both cytoplasmic and nuclear staining was observed, but the distribution of staining was predominantly nuclear (Figure 1C). Stromal nuclei and cytoplasm were also stained positive. Low-to-moderate extent of staining was observed in both the epithelium and stroma of benign and borderline tumours.

#### Grades 1, 2 and 3 tumours

**Figure 2:**
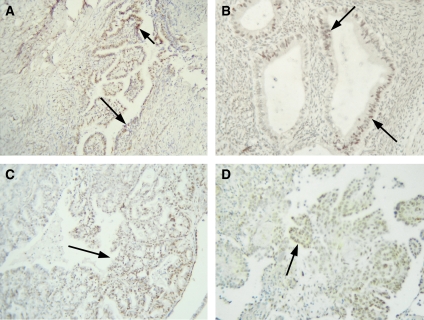
Expression of PPAR*β* in grades 1, 2 and 3 ovarian tumours. (**A**) Grade 1 endometrioid; (**B**) grade 1 mucinuous; (**C**) grade 2 endometrioid and (**D**) grade 3 serous tumours. Arrows in each tumour illustrate positive nuclear PPAR*β* staining of the scattered epithelium.

Grade 1 ovarian tumours exhibited less staining than their benign and borderline counterparts (Figure 2A and B). Four out of nine tumours exhibited <50% epithelial staining, and in some tumours staining as little as 15% was observed (Table 1). Staining of the epithelial cells was mostly nuclear but some cytoplasmic staining was also evident. Stromal staining was also reduced with four samples demonstrating <10% staining (Table 1). Grades 2 and 3 tumours also showed less staining than benign and borderline tumours (Figure 2C and D). Staining was mostly confined to epithelial cells with minimum stromal staining. Staining was predominantly nuclear, although some degree of cytoplasmic staining was also evident. Nuclear staining of infiltrating macrophages was also evident in some sections. The staining intensity in both the epithelium and stroma of grades 1, 2 and 3 tumours was weak.

### Immunohistochemical expression of PDK1 in ovarian tissues

#### Normal ovaries and benign tumours

**Figure 3:**
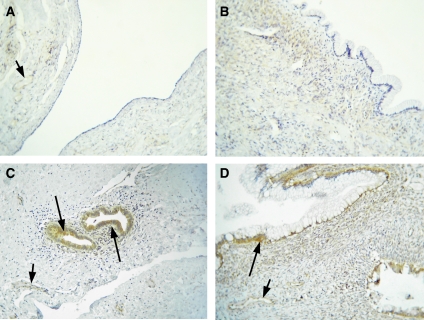
Expression of PDK1 in normal ovaries, benign and borderline ovarian tumours. Archival ovarian tissues were stained by the methods discussed in the Materials and methods section. (**A**) Normal ovary, no staining of the epithelium was evident, but very weak staining of the blood vessel is indicated by a short arrow. (**B**) Benign mucinous ovarian tumour lacking PDK1 expression in the epithelium; (**C**) benign serous ovarian tumour illustrating strong PDK1 staining of the inclusion cysts (long arrow) and blood vessels (short arrow). (**D**) Borderline mucinous ovarian tumour, long arrow displays positive PDK1 staining of the epithelium, whereas short arrow illustrates weak staining of the blood vessels.

No staining of PDK1 was evident in 9 out of 10 normal ovaries used in the study (Table 2, Figure 3A). Very weak cytoplasmic staining of the epithelium was present in one ovary. Four out of 10 benign tumours showed negative PDK1 staining (Table 2, Figure 3B), whereas weak cytoplasmic staining was evident in 5 out of 10 benign tumours. Only one serous benign tumour demonstrated moderate staining for PDK1, and it was mostly confined to the inclusion cysts present in the section (Figure 3C). Stromal staining was not evident in either normal ovaries or in benign tumours. Staining of the blood vessels was evident in the sections that contained them.

#### Borderline and grades 1–3 tumours

Ten pre-malignant borderline ovarian tumours revealed low (*n*=5), moderate (*n*=2) and high (*n*=3) PDK1 expression (Table 2 and Figure 3D). Most of the tumours displayed diffuse cytoplasmic staining with few sites displaying membrane staining confined to the epithelium. High extent of staining was observed in serous ovarian tumours. In some sections, weak staining of the reactive stroma adjacent to the epithelium was also evident (Figure 3D). Weak staining of the blood vessels was also present in some sections (Figure 3D).

**Figure 4:**
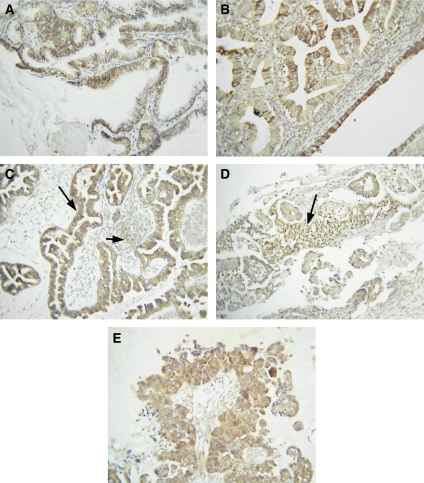
Expression of PDK1 in grades 1, 2 and 3 ovarian tumours. (**A**) Grade 1 mucinuous and (**B**) grade 1 endometrioid tumours displaying strong PDK1 staining. (**C**) Grade 2 serous tumour, long arrow indicates strong epithelial staining and short arrow exhibits staining of the reactive stroma. (**D**) Grade 3 serous tumour displaying scattered epithelial tumour cells positively stained for PDK1. (**E**) Grade 3 serous ovarian tumour strongly positive for PDK1 staining.

Nine grade 1 tumours were available for examination. Two of the grade 1 tumours displayed week staining, whereas moderate staining was evident in five grade 1 tumours with high extent of staining in two endometrioid grade 1 tumours. In case of grade 1 tumours, both cytoplasmic and membrane staining were displayed (Figure 4A and B), with the degree of membrane staining more prevalent than in borderline tumours. Staining was mostly confined to the epithelium, and some degree of the staining of the reactive stroma was evident in the sections that contained them (Figure 4). Twenty-two grades 2 and 3 ovarian tumours were examined for PDK1 expression. Out of these, only two grade 2 tumours displayed weak staining, whereas six displayed moderate staining and the rest (*n*=14) displayed high extent of staining. The staining pattern of grades 2 and 3 tumours was similar to that of grade 1 tumours (Figure 4C–E), with the occurrence of more membrane staining of the epithelial cells compared to the diffuse cytoplasmic staining. The extent of staining ranged from the majority of tumour cells to scattered positive cells in some sections. Multiple nests of cells displayed diffuse cytoplasmic staining that ranged in intensity. In some sections, the intercellular membranes were highlighted in some areas, whereas in others there was more involvement of the cytoplasm.

**Figure 5:**
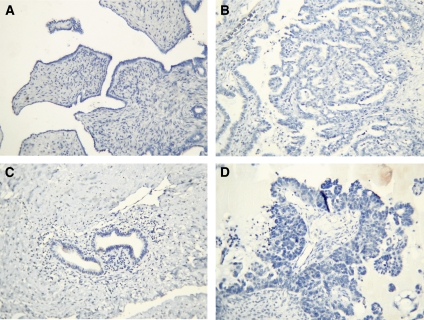
Negative IgG controls for PPAR*β* (**A** and **B**) benign serous and grade 3 serous ovarian tumours; for PDK1 (**C** and **D**) benign serous and grade 3 serous ovarian tumour.

None of the tissues showed any positive staining with the control IgG antibodies (Figure 5).

#### Statistical analysis

PPAR*β*: The extent of staining in the epithelium and stroma was significantly different within normal and different histological grades of cancer (*χ*^2^=59.25, d.f.=25, *P*<0.001; *χ*^2^=64.48, d.f.=25, *P*<0.001). The extent of epithelial and stromal staining between normal ovaries and grades 1, 2 and 3 tumours was also significantly different (*χ*^2^=11.28, d.f.=4, *P*<0.05; *χ*^2^=16.15, d.f.=4, *P*<0.005). There was no significant difference in the extent of epithelial staining between normal *vs* benign/borderline groups. However, significance was observed with respect to stromal staining (*χ*^2^=11.58, d.f.=4, *P*<0.05) between normal and benign/borderline tumours. Within the tumours, the extent of staining in the epithelium and stroma was significantly different in benign and borderline tumours compared with grades 1, 2 and 3 tumours (*χ*^2^=25.53, d.f.=5, *P*<0.001; *χ*^2^=42.80, d.f.=5, *P*<0.001).

PDK1: The expression of immunoreactive PDK1 was present in only 10% of normal ovaries and 60% of benign ovarian tumours. The extent of staining in the epithelium was significantly different within normal and different histological grades of cancer (*χ*^2^=71.32, d.f.=25, *P*<0.001). Within the tumours, the extent of staining in the epithelium was significantly different between benign/borderline *vs* grades 1, 2 and 3 groups (*χ*^2^=22.45, d.f.=5, *P*<0.001); however, no significant difference was observed between benign and borderline tumours or grades 1, 2 and 3 tumours.

## DISCUSSION

We have recently demonstrated that the cytoplasmic and nuclear expression of PPAR*γ* increases progressively with the progression of ovarian carcinoma (Zhang *et al*, 2005). We have also demonstrated enhanced expression of ILK, a putative target gene of PPAR*β*, in high-grade ovarian tumours (Ahmed *et al*, 2003) and the presence of cell-free irILK in the serum and ascites of ovarian cancer patients (Ahmed *et al*, 2004). On the basis of these observations, we hypothesised that the expression of PPAR*β* and its downstream target PDK1 will also change with the progression of ovarian carcinoma. In this study, we report the differential expression of PPAR*β* and PDK1 in normal human ovaries and ovarian tumours of different histological grades and subtypes.

Normal ovarian tissues expressed PPAR*β*. The expression was moderate, predominantly nuclear and localised to both the epithelium and stroma. Moderate–to-strong expression of PPAR*β* was observed in benign and borderline tumours with the staining being predominantly nuclear and localised to the epithelium and stroma. In some benign tumours, staining of inclusion cysts was also evident (Figure 1B). In grades 1, 2 and 3 tumours, scattered staining of the epithelial cells within the stroma was observed (Figure 2A–D). Stromal staining was less evident, and in some tumours <10% of the stromal cells were stained. The extent of staining ranged from negative to moderate with few high-grade tumours demonstrating no epithelial or stromal staining. In some high-grade tumours, infiltrating macrophages often demonstrated moderate staining, consistent with the relatively high expression of PPAR*β* in macrophages (Feige *et al*, 2006). These results suggest that PPAR*β* is expressed in the epithelium and stroma of normal ovaries, benign, borderline and low- to high-grade ovarian tumours, but compared to benign and borderline tumours, there is a declining pattern of expression in high-grade ovarian tumours. The expression of PPAR*β* has been demonstrated previously in the theca and stromal component of normal ovaries (Froment *et al*, 2006). Considering that the expression and activation of PPAR*β* is usually triggered by inflammation (Tan *et al*, 2001) and that normal ovaries undergo inflammation-mediated responses even immediately after menopause (due to high levels of gonadotrophins), the persistent expression of PPAR*β* in the epithelial and stromal component of normal ovaries of the control group used in the study is not surprising. A recent study, however, has demonstrated the expression of PPAR*β* in mouse models of ovarian tumours as well as the that PPAR*β* mRNA and protein to be expressed in tumours generated *in vivo* by mouse ovarian cancer cell lines, whereas low-to-undetectable levels of PPAR*β* were expressed in normal mouse ovaries and non-tumourous ovarian samples (Daikoku *et al*, 2007). The lack of PPAR*β* expression in normal mouse ovaries and non-tumorous ovarian samples may be attributed to differences in the techniques (*in situ* hybridisation and Western blot compared to immunohistochemistry) used in both studies or may be the result of a cell type-specific response generated in immunocompromised mice by oncogene (*Kras*, *cmyc*, *RCAS*, *Akt*, and so on) carrying virus-transfected mouse ovarian cell lines (Daikoku *et al*, 2007).

The relatively high expression of PPAR*β* in differentiated benign and borderline ovarian tumours compared to high-grade tumours may implicate its known role in differentiation described previously in keratinocytes, colonocytes and breast cancer cells (Di-Poi *et al*, 2005; Aung *et al*, 2006). Besides keratinocytes, PPAR*β* has also been shown to regulate the differentiation of primary macrophages or a monocyte/macrophage cell line (Vosper *et al*, 2001). The activation of PPAR*β* using a selective agonist promotes oligodendrocyte differentiation in a mouse cell culture model (Saluja *et al*, 2001) consistent with the myelination defect of the *corpus callosum* in PPAR*β* null mice (Peters *et al*, 2000). Peroxisome proliferator-activated receptor *β* also contributes to adipose tissue differentiation as demonstrated by the decrease in fat mass in PPAR*β* null mice (Peters *et al*, 2000; Barak *et al*, 2002). In cancers, ligand activation of PPAR*β* has been shown to attenuate colon carcinogenesis (Harman *et al*, 2004; Marin *et al*, 2006), and decreased PPAR*β* expression has been displayed by microarray analysis in high-grade tumour samples compared to control tissues, suggesting that decreased expression of PPAR*β* may be linked to an increase in the loss of differentiation required for the progression of colon malignancies (Notterman *et al*, 2001). These studies have similarities with our study, and attenuated expression of PPAR*β* in high-grade ovarian carcinomas may implicate the inability of the tumours to maintain differentiation with the progression of the malignancy.

The involvement of PPAR*β* in colorectal cancer is complex and seems to be regulated by pathways other than those controlling cellular differentiation. A recent report showed that the expression and activation of PPAR*β* is increased in rat intestinal cells by the overexpression of activated Kras oncogene (Shao *et al*, 2002). In another colorectal cancer cell xenograft model, the absence of PPAR*β* decreased tumorigenicity, indicating that the expression of PPAR*β* may be involved with colon tumorigenesis (Park *et al*, 2001a). In contrast, APC^Min^PPAR*β* null mouse showed no requirement for PPAR*β* for polyp formation (Barak *et al*, 2002), indicating that the increased activity of PPAR*β* on an APC-null background might be one of the factors supporting colorectal tumorigenesis (Michalik *et al*, 2004). Hence, the status of PPAR*β* expression in colorectal cancer is controversial and whether it is regulated by the APC or the K*ras* oncogene yet remains to be determined.

Some recent studies have shown PPAR*β* as a potent inhibitor of PPAR*α*- and PPAR*γ*-activated transcription (Shi *et al*, 2002). The induced expression of PPAR*β* in 3T3-PPAR*γ* adipocytes inhibited PPAR*γ*-induced gene expression and adipogenesis (Bastie *et al*, 1999). The increased expression of PPAR*β* in hepatic stellate cells induced increased proliferation and fibrogenesis with concomitant decreased expression of PPAR*γ* (Hellemans *et al*, 2003), suggesting that PPAR*β* and PPAR*γ* may be inversely regulated. These studies are consistent with our previous study where we have reported enhanced expression of PPAR*γ* in high-grade ovarian tumours compared with benign and borderline ovarian tumours (Zhang *et al*, 2005). Hence, a balance of PPAR*β* and PPAR*γ* expression and activation may be needed to regulate ovarian tumour differentiation and metastases.

In contrast to PPAR*β* expression, PDK1 expression was absent in 90% of normal ovaries, and low expression of PDK1 was evident in only 1 out of 10 normal ovaries. In the case of benign ovarian tumours, <50% were negative for PDK1 expression, whereas the rest displayed low-to-moderate PDK1 expression confined to the cytoplasm and membranes of the epithelium. Nearly 50% of borderline ovarian tumours displayed low expression of PDK1 and the other 50% demonstrated moderate-to-high expression. There was a gradual increase in the expression of PDK1 with increasing grades of ovarian tumours, with the bulk of the high-grade tumours demonstrating high expression of cytoplasmic and membrane-bound PDK1. The apparent increase in PDK1 expression in high-grade ovarian carcinomas is consistent with our previously reported parallel increase in ILK expression with increasing grade of ovarian carcinomas (Ahmed *et al*, 2003). A similar trend of heightened expression of both ILK and PDK1 in high-grade ovarian tumours may suggest enhanced activation of upstream PI-3 kinase and/or PPAR*β* cascades required for tumour progression. The overexpression of growth factor receptors (e.g., Met receptor, EGFR, and so on) and their downstream signalling such as PI-3 kinase has been reported in high-grade ovarian tumours (Stadlmann *et al*, 2006; Sawada *et al*, 2007). As the expression of PPAR*β* does not get elevated in high-grade ovarian tumours, it can be suggested that sustained activation of the PI-3 kinase pathway either due to aberrant activation of growth factor receptors or loss of PTEN function may be required to maintain elevated expression and activation of ILK, PDK1 and the downstream Akt and Wnt pathways in advanced ovarian tumours.

The transforming ability of PDK1 *in vitro* and *in vivo* is mediated by PKC*α* and is linked to c-myc function and the expression of caveolin-1, an integral protein component of caveolae known for its role as a tumour suppressor (Xie *et al*, 2003). The forced expression of PDK1 and PKC*α* in mammary epithelial cells results in the upregulation of c-Myc with correlated downregulation of caveolin-1 expression and gain of transformation (Xie *et al*, 2003). Overexpression and amplification of c-myc gene copy number have been reported in ovarian tumours (Dimova *et al*, 2006), and the inhibition or depletion of PKC*α* activity has been shown to inhibit drug resistance (Masanek *et al*, 2002) or sensitise ovarian cancer cells to platinum drugs (Isonishi *et al*, 2000). Moreover, caveolin-1 expression, which has been demonstrated in normal and benign ovarian tumours, has been shown to recede in high-grade ovarian carcinomas, and forced expression of caveolin-1 in ovarian cancer cells resulted in loss of cell survival, consistent with the tumour-suppressing role of caveolin-1 described for other cancers (Wiechen *et al*, 2001). Taken together, these observations suggest a correlative link between PDK1, c-myc, PKC*α* and caveolin-1 in ovarian tumours and are consistent with those observed in mammary epithelial cell models (Xie *et al*, 2003, 2006).

In conclusion, a better understanding of PPAR*β* function as well as its activation and repression in *in vitro* and *in vivo* models of ovarian cancer is needed to ascertain its role as a potential therapeutic target. On the other hand, the lack of PDK1 expression in normal ovaries, its weak expression in benign tumours and its elevated expression in pre-malignant and low- to high-grade ovarian carcinomas provide compelling evidence of its oncogeneic role in ovarian cancer progression. Hence, like other cancers, PDK1 may be a preferred molecular target for sensitising ovarian cancer cells to chemotherapeutic agents (Feldman *et al*, 2005).

## Figures and Tables

**Table 1:** The extent of PPAR*β* expression in normal ovaries and ovarian tumours

**Histology**	**Total number of tissues**	**Extent of staining in the epithelium (number of tissues)**	**Extent of staining in the stroma (number of tissues)**
Normal	10	1(1), 2(1), 3 (4), 4(4)	1(1), 2(5), 3(4)
Benign	10	1(3), 2(1), 3(1), 4(2), 5(3)	2(2), 3(3), 4(2), 5(3)
Borderline	10	3(1), 4(5), 5(4)	3(5), 4(4), 5(1)
Grade 1	9	1(3), 2(1), 3(5)	0(4), 1(3), 2(2)
Grade 2	10	0 (3), 1 (2), 3 (4), 4(1)	0(5), 1(3), 2(2)
Grade 3	10	0 (1), 1 (6), 2 (3)	0(5), 1(3), 2(2)
Total	59		

PPAR*β*, peroxisome proliferator-activated receptor *β*.

The extent of PPAR*β* expression was scored as 0 (⩽10%), 1 (⩾11–25%), 2 (⩾26–50%), 3 (⩾51–75%), 4(⩾76–90%) and 5 (⩾91–100%) immunoreactivity. Values in the parentheses indicate number of tissues in each category.

**Table 2:** The extent of PDK1 expression in normal ovaries and ovarian tumours

**Histology**	**Total number of tissues**	**Extent of staining in the epithelium**
Normal	10	0 (9), 1 (1)
Benign	10	0 (4), 1 (3), 2 (2), 4 (1)
Borderline	10	1 (2), 2 (3), 3 (2), 4 (3)
Grade 1	9	2 (2), 3 (5), 4 (2)
Grade 2	11	2 (2), 3 (4), 4 (4), 5 (1)
Grade 3	11	3 (2), 4 (7), 5 (2)
Total	61	

The extent of PDK1 expression was scored as 0 (⩽10%), 1 (⩾11–25%), 2 (⩾26–50%), 3 (⩾51–75%), 4(⩾76–90%) and 5 (⩾91–100%) immunoreactivity. Values in the parentheses indicate number of tissues in each category.

## References

[bib1] Ahmed N, Oliva K, Rice GE, Quinn MA (2004) Cell-free 59 kDa immunoreactive integrin-linked kinase: a novel marker for ovarian carcinoma. Clin Cancer Res 10: 2415–2420 15073119 10.1158/1078-0432.ccr-03-0042

[bib2] Ahmed N, Pansino F, Clyde R, Murthi P, Quinn MA, Rice GE, Agrez MV, Mok S, Baker MS (2002a) Overexpression of alpha(v)beta6 integrin in serous epithelial ovarian cancer regulates extracellular matrix degradation via the plasminogen activation cascade. Carcinogenesis 23: 237–244 11872628 10.1093/carcin/23.2.237

[bib3] Ahmed N, Riley C, Oliva K, Rice G, Quinn M (2005) Ascites induces modulation of alpha6beta1 integrin and urokinase plasminogen activator receptor expression and associated functions in ovarian carcinoma. Br J Cancer 92: 1475–1485 15798771 10.1038/sj.bjc.6602495 PMC2362012

[bib4] Ahmed N, Riley C, Oliva K, Stutt E, Rice GE, Quinn MA (2003) Integrin-linked kinase expression increases with ovarian tumour grade and is sustained by peritoneal tumour fluid. J Pathol 201: 229–237 14517840 10.1002/path.1441

[bib5] Ahmed N, Riley C, Rice GE, Quinn MA, Baker MS (2002b) Alpha(v)beta(6) integrin-A marker for the malignant potential of epithelial ovarian cancer. J Histochem Cytochem 50: 1371–1380 12364570 10.1177/002215540205001010

[bib6] Alessi DR, James SR, Downes CP, Holmes AB, Gaffney PR, Reese CB, Cohen P (1997) Characterization of a 3-phosphoinositide-dependent protein kinase which phosphorylates and activates protein kinase Balpha. Curr Biol 7: 261–269 9094314 10.1016/s0960-9822(06)00122-9

[bib7] Alessi DR, Kozlowski MT, Weng QP, Morrice N, Avruch J (1998) 3-Phosphoinositide-dependent protein kinase 1 (PDK1) phosphorylates and activates the p70 S6 kinase *in vivo* and *in vitro*. Curr Biol 8: 69–81 9427642 10.1016/s0960-9822(98)70037-5

[bib8] Arboleda MJ, Lyons JF, Kabbinavar FF, Bray MR, Snow BE, Ayala R, Danino M, Karlan BY, Slamon DJ (2003) Overexpression of AKT2/protein kinase Bbeta leads to up-regulation of beta1 integrins, increased invasion, and metastasis of human breast and ovarian cancer cells. Cancer Res 63: 196–206 12517798

[bib9] Aung CS, Faddy HM, Lister EJ, Monteith GR, Roberts-Thomson SJ (2006) Isoform specific changes in PPAR alpha and beta in colon and breast cancer with differentiation. Biochem Biophys Res Commun 340: 656–660 16378595 10.1016/j.bbrc.2005.12.061

[bib10] Barak Y, Liao D, He W, Ong ES, Nelson MC, Olefsky JM, Boland R, Evans RM (2002) Effects of peroxisome proliferator-activated receptor delta on placentation, adiposity, and colorectal cancer. Proc Natl Acad Sci USA 99: 303–308 11756685 10.1073/pnas.012610299 PMC117556

[bib11] Bastie C, Holst D, Gaillard D, Jehl-Pietri C, Grimaldi PA (1999) Expression of peroxisome proliferator-activated receptor PPARdelta promotes induction of PPARgamma and adipocyte differentiation in 3T3C2 fibroblasts. J Biol Chem 274: 21920–21925 10419513 10.1074/jbc.274.31.21920

[bib12] Bastie C, Luquet S, Holst D, Jehl-Pietri C, Grimaldi PA (2000) Alterations of peroxisome proliferator-activated receptor delta activity affect fatty acid-controlled adipose differentiation. J Biol Chem 275: 38768–38773 10991946 10.1074/jbc.M006450200

[bib13] Bayascas JR, Leslie NR, Parsons R, Fleming S, Alessi DR (2005) Hypomorphic mutation of PDK1 suppresses tumorigenesis in PTEN(±) mice. Curr Biol 15: 1839–1846 16243031 10.1016/j.cub.2005.08.066

[bib14] Burdick AD, Kim DJ, Peraza MA, Gonzalez FJ, Peters JM (2006) The role of peroxisome proliferator-activated receptor-beta/delta in epithelial cell growth and differentiation. Cell Signal 18: 9–20 16109478 10.1016/j.cellsig.2005.07.009

[bib15] Daikoku T, Tranguch S, Chakrabarty A, Wang D, Khabele D, Orsulic S, Morrow JD, Dubois RN, Dey SK (2007) Extracellular signal-regulated kinase is a target of cyclooxygenase-1-peroxisome proliferator-activated receptor-delta signaling in epithelial ovarian cancer. Cancer Res 67: 5285–5292 17545608 10.1158/0008-5472.CAN-07-0828

[bib16] Dimova I, Raitcheva S, Dimitrov R, Doganov N, Toncheva D (2006) Correlations between c-myc gene copy-number and clinicopathological parameters of ovarian tumours. Eur J Cancer 42: 674–679 16458500 10.1016/j.ejca.2005.11.022

[bib17] Dinulescu DM, Ince TA, Quade BJ, Shafer SA, Crowley D, Jacks T (2005) Role of K-ras and Pten in the development of mouse models of endometriosis and endometrioid ovarian cancer. Nat Med 11: 63–70 15619626 10.1038/nm1173

[bib18] Di-Poi N, Michalik L, Tan NS, Desvergne B, Wahli W (2003) The anti-apoptotic role of PPARbeta contributes to efficient skin wound healing. J Steroid Biochem Mol Biol 85: 257–265 12943711 10.1016/s0960-0760(03)00215-2

[bib19] Di-Poi N, Ng CY, Tan NS, Yang Z, Hemmings BA, Desvergne B, Michalik L, Wahli W (2005) Epithelium–mesenchyme interactions control the activity of peroxisome proliferator-activated receptor beta/delta during hair follicle development. Mol Cell Biol 25: 1696–1712 15713628 10.1128/MCB.25.5.1696-1712.2005 PMC549363

[bib20] Di-Poi N, Tan NS, Michalik L, Wahli W, Desvergne B (2002) Antiapoptotic role of PPARbeta in keratinocytes via transcriptional control of the Akt1 signaling pathway. Mol Cell 10: 721–733 12419217 10.1016/s1097-2765(02)00646-9

[bib21] Dutil EM, Toker A, Newton AC (1998) Regulation of conventional protein kinase C isozymes by phosphoinositide-dependent kinase 1 (PDK-1). Curr Biol 8: 1366–1375 9889098 10.1016/s0960-9822(98)00017-7

[bib22] Feige JN, Gelman L, Michalik L, Desvergne B, Wahli W (2006) From molecular action to physiological outputs: peroxisome proliferator-activated receptors are nuclear receptors at the crossroads of key cellular functions. Prog Lipid Res 45: 120–159 16476485 10.1016/j.plipres.2005.12.002

[bib23] Feldman RI, Wu JM, Polokoff mA, Kochanny MJ, Dinter H, Zhu D, Biroc SL, Alicke B, Bryant J, Yuan S, Buckman BO, Lentz D, Ferrer M, Whitlow M, Adler M, Finster S, Chang Z, Arnaiz DO (2005) Novel small molecule inhibitors of 3-phosphoinositide-dependent kinase-1. J Biol Chem 280: 19867–19874 15772071 10.1074/jbc.M501367200

[bib24] Flynn P, Mellor H, Casamassima A, Parker PJ (2000a) Rho GTPase control of protein kinase C-related protein kinase activation by 3-phosphoinositide-dependent protein kinase. J Biol Chem 275: 11064–11070 10753910 10.1074/jbc.275.15.11064

[bib25] Flynn P, Wongdagger M, Zavar M, Dean NM, Stokoe D (2000b) Inhibition of PDK-1 activity causes a reduction in cell proliferation and survival. Curr Biol 10: 1439–1442 11102805 10.1016/s0960-9822(00)00801-0

[bib26] Fresno Vara JA, Casado E, de Castro J, Cejas P, Belda-Iniesta C, Gonzalez-Baron M (2004) PI3K/Akt signalling pathway and cancer. Cancer Treat Rev 30: 193–204 15023437 10.1016/j.ctrv.2003.07.007

[bib27] Froment P, Gizard F, Defever D, Staels B, Dupont J, Monget P (2006) Peroxisome proliferator-activated receptors in reproductive tissues: from gametogenesis to parturition. J Endocrinol 189: 199–209 16648288 10.1677/joe.1.06667

[bib28] Glinghammar B, Skogsberg J, Hamsten A, Ehrenborg E (2003) PPARdelta activation induces COX-2 gene expression and cell proliferation in human hepatocellular carcinoma cells. Biochem Biophys Res Commun 308: 361–368 12901877 10.1016/s0006-291x(03)01384-6

[bib29] Gupta RA, Wang D, Katkuri S, Wang H, Dey SK, DuBois RN (2004) Activation of nuclear hormone receptor peroxisome proliferator-activated receptor-delta accelerates intestinal adenoma growth. Nat Med 10: 245–247 14758356 10.1038/nm993

[bib30] Han S, Ritzenthaler JD, Wingerd B, Roman J (2005) Activation of peroxisome proliferator-activated receptor beta/delta (PPARbeta/delta) increases the expression of prostaglandin E2 receptor subtype EP4. The roles of phosphatidylinositol 3-kinase and CCAAT/enhancer-binding protein beta. J Biol Chem 280: 33240–33249 16061473 10.1074/jbc.M507617200

[bib31] Harman FS, Nicol CJ, Marin HE, Ward JM, Gonzalez FJ, Peters JM (2004) Peroxisome proliferator-activated receptor-delta attenuates colon carcinogenesis. Nat Med 10: 481–483 15048110 10.1038/nm1026

[bib32] Hellemans K, Michalik L, Dittie A, Knorr A, Rombouts K, De Jong J, Heirman C, Quartier E, Schuit F, Wahli W, Geerts A (2003) Peroxisome proliferator-activated receptor-beta signaling contributes to enhanced proliferation of hepatic stellate cells. Gastroenterology 124: 184–201 12512042 10.1053/gast.2003.50015

[bib33] Isonishi S, Ohkawa K, Tanaka T, Howell SB (2000) Depletion of protein kinase C (PKC) by 12-O-tetradecanoylphorbol-13-acetate (TPA) enhances platinum drug sensitivity in human ovarian carcinoma cells. Br J Cancer 82: 34–38 10638963 10.1054/bjoc.1999.0873 PMC2363215

[bib34] Jaeckel EC, Raja S, Tan J, Das SK, Dey SK, Girod DA, Tsue TT, Sanford TR (2001) Correlation of expression of cyclooxygenase-2, vascular endothelial growth factor, and peroxisome proliferator-activated receptor delta with head and neck squamous cell carcinoma. Arch Otolaryngol Head Neck Surg 127: 1253–1259 11587608 10.1001/archotol.127.10.1253

[bib35] Kim DJ, Akiyama TE, Harman FS, Burns AM, Shan W, Ward JM, Kennett MJ, Gonzalez FJ, Peters JM (2004) Peroxisome proliferator-activated receptor beta (delta)-dependent regulation of ubiquitin C expression contributes to attenuation of skin carcinogenesis. J Biol Chem 279: 23719–23727 15033975 10.1074/jbc.M312063200

[bib36] King CC, Gardiner EM, Zenke FT, Bohl BP, Newton AC, Hemmings BA, Bokoch GM (2000) p21-Activated kinase (PAK1) is phosphorylated and activated by 3-phosphoinositide-dependent kinase-1 (PDK1). J Biol Chem 275: 41201–41209 10995762 10.1074/jbc.M006553200

[bib37] Le Good JA, Ziegler WH, Parekh DB, Alessi DR, Cohen P, Parker PJ (1998) Protein kinase C isotypes controlled by phosphoinositide 3-kinase through the protein kinase PDK1. Science 281: 2042–2045 9748166 10.1126/science.281.5385.2042

[bib38] Marin HE, Peraza MA, Billin AN, Willson TM, Ward JM, Kennett MJ, Gonzalez FJ, Peters JM (2006) Ligand activation of peroxisome proliferator-activated receptor beta inhibits colon carcinogenesis. Cancer Res 66: 4394–4401 16618765 10.1158/0008-5472.CAN-05-4277

[bib39] Masanek U, Stammler G, Volm M (2002) Modulation of multidrug resistance in human ovarian cancer cell lines by inhibition of P-glycoprotein 170 and PKC isoenzymes with antisense oligonucleotides. J Exp Ther Oncol 2: 37–41 12415618 10.1046/j.1359-4117.2002.01004.x

[bib40] Michalik L, Desvergne B, Wahli W (2003) Peroxisome proliferator-activated receptors beta/delta: emerging roles for a previously neglected third family member. Curr Opin Lipidol 14: 129–135 12642780 10.1097/00041433-200304000-00003

[bib41] Michalik L, Desvergne B, Wahli W (2004) Peroxisome-proliferator-activated receptors and cancers: complex stories. Nat Rev Cancer 4: 61–70 14708026 10.1038/nrc1254

[bib42] Miled N, Yan Y, Hon WC, Perisic O, Zvelebil M, Inbar Y, Schneidman-Duhovny D, Wolfson HJ, Backer JM, Williams RL (2007) Mechanism of two classes of cancer mutations in the phosphoinositide 3-kinase catalytic subunit. Science 317: 239–242 17626883 10.1126/science.1135394

[bib43] Nahle Z (2004) PPAR trilogy from metabolism to cancer. Curr Opin Clin Nutr Metab Care 7: 397–402 15192441 10.1097/01.mco.0000134360.30911.bb

[bib44] Notterman DA, Alon U, Sierk AJ, Levine AJ (2001) Transcriptional gene expression profiles of colorectal adenoma, adenocarcinoma, and normal tissue examined by oligonucleotide arrays. Cancer Res 61: 3124–3130 11306497

[bib45] Park BH, Vogelstein B, Kinzler KW (2001a) Genetic disruption of PPARdelta decreases the tumorigenicity of human colon cancer cells. Proc Natl Acad Sci USA 98: 2598–2603 11226285 10.1073/pnas.051630998 PMC30184

[bib46] Park BK, Zeng X, Glazer RI (2001b) Akt1 induces extracellular matrix invasion and matrix metalloproteinase-2 activity in mouse mammary epithelial cells. Cancer Res 61: 7647–7653 11606407

[bib47] Peters JM, Lee SS, Li W, Ward JM, Gavrilova O, Everett C, Reitman ML, Hudson LD, Gonzalez FJ (2000) Growth, adipose, brain, and skin alterations resulting from targeted disruption of the mouse peroxisome proliferator-activated receptor beta(delta). Mol Cell Biol 20: 5119–5128 10866668 10.1128/mcb.20.14.5119-5128.2000 PMC85961

[bib48] Planavila A, Rodriguez-Calvo R, Jove M, Michalik L, Wahli W, Laguna JC, Vazquez-Carrera M (2005) Peroxisome proliferator-activated receptor beta/delta activation inhibits hypertrophy in neonatal rat cardiomyocytes. Cardiovasc Res 65: 832–841 15721863 10.1016/j.cardiores.2004.11.011

[bib49] Pullen N, Dennis PB, Andjelkovic M, Dufner A, Kozma SC, Hemmings BA, Thomas G (1998) Phosphorylation and activation of p70s6k by PDK1. Science 279: 707–710 9445476 10.1126/science.279.5351.707

[bib50] Reed KR, Sansom OJ, Hayes AJ, Gescher AJ, Winton DJ, Peters JM, Clarke AR (2004) PPARdelta status and Apc-mediated tumourigenesis in the mouse intestine. Oncogene 23: 8992–8996 15480419 10.1038/sj.onc.1208143

[bib51] Roymans D, Slegers H (2001) Phosphatidylinositol 3-kinases in tumor progression. Eur J Biochem 268: 487–498 11168386 10.1046/j.1432-1327.2001.01936.x

[bib52] Saluja I, Granneman JG, Skoff RP (2001) PPAR delta agonists stimulate oligodendrocyte differentiation in tissue culture. Glia 33: 191–204 11241737

[bib53] Sawada K, Radjabi AR, Shinomiya N, Kistner E, Kenny H, Becker AR, Turkyilmaz MA, Salgia R, Yamada SD, Vande Woude GF, Tretiakova MS, Lengyel E (2007) c-Met overexpression is a prognostic factor in ovarian cancer and an effective target for inhibition of peritoneal dissemination and invasion. Cancer Res 67: 1670–1679 17308108 10.1158/0008-5472.CAN-06-1147

[bib54] Shao J, Sheng H, DuBois RN (2002) Peroxisome proliferator-activated receptors modulate K-Ras-mediated transformation of intestinal epithelial cells. Cancer Res 62: 3282–3288 12036946

[bib55] Shi Y, Hon M, Evans RM (2002) The peroxisome proliferator-activated receptor delta, an integrator of transcriptional repression and nuclear receptor signaling. Proc Natl Acad Sci USA 99: 2613–2618 11867749 10.1073/pnas.052707099 PMC122396

[bib56] Silverberg SG (2000) Histopathologic grading of ovarian carcinoma: a review and proposal. Int J Gynecol Pathol 19: 7–15 10638449 10.1097/00004347-200001000-00003

[bib57] Somasiri A, Howarth A, Goswami D, Dedhar S, Roskelley CD (2001) Overexpression of the integrin-linked kinase mesenchymally transforms mammary epithelial cells. J Cell Sci 114: 1125–1136 11228156 10.1242/jcs.114.6.1125

[bib58] Stadlmann S, Gueth U, Reiser U, Diener PA, Zeimet AG, Wight E, Mirlacher M, Sauter G, Mihatsch MJ, Singer G (2006) Epithelial growth factor receptor status in primary and recurrent ovarian cancer. Mod Pathol 19: 607–610 16554736 10.1038/modpathol.3800575

[bib59] Stiles B, Gilman V, Khanzenzon N, Lesche R, Li A, Qiao R, Liu X, Wu H (2002) Essential role of AKT-1/protein kinase B alpha in PTEN-controlled tumorigenesis. Mol Cell Biol 22: 3842–3851 11997518 10.1128/MCB.22.11.3842-3851.2002 PMC133830

[bib60] Tan NS, Michalik L, Desvergne B, Wahli W (2003) Peroxisome proliferator-activated receptor (PPAR)-beta as a target for wound healing drugs: what is possible? Am J Clin Dermatol 4: 523–530 12862494 10.2165/00128071-200304080-00001

[bib61] Tan NS, Michalik L, Noy N, Yasmin R, Pacot C, Heim M, Fluhmann B, Desvergne B, Wahli W (2001) Critical roles of PPAR beta/delta in keratinocyte response to inflammation. Genes Dev 15: 3263–3277 11751632 10.1101/gad.207501 PMC312855

[bib62] Toker A, Newton AC (2000) Cellular signaling: pivoting around PDK-1. Cell 103: 185–188 11057891 10.1016/s0092-8674(00)00110-0

[bib63] Tong BJ, Tan J, Tajeda L, Das SK, Chapman JA, DuBois RN, Dey SK (2000) Heightened expression of cyclooxygenase-2 and peroxisome proliferator-activated receptor-delta in human endometrial adenocarcinoma. Neoplasia 2: 483–490 11228540 10.1038/sj.neo.7900119 PMC1508090

[bib64] Vanhaesebroeck B, Alessi DR (2000) The PI3K–PDK1 connection: more than just a road to PKB. Biochem J 346(Pt 3): 561–576 10698680 PMC1220886

[bib65] Vosper H, Patel L, Graham TL, Khoudoli GA, Hill A, Macphee CH, Pinto I, Smith SA, Suckling KE, Wolf CR, Palmer CN (2001) The peroxisome proliferator-activated receptor delta promotes lipid accumulation in human macrophages. J Biol Chem 276: 44258–44265 11557774 10.1074/jbc.M108482200

[bib66] Wiechen K, Diatchenko L, Agoulnik A, Scharff KM, Schober H, Arlt K, Zhumabayeva B, Siebert PD, Dietel M, Schafer R, Sers C (2001) Caveolin-1 is down-regulated in human ovarian carcinoma and acts as a candidate tumor suppressor gene. Am J Pathol 159: 1635–1643 11696424 10.1016/S0002-9440(10)63010-6 PMC1867061

[bib67] Xie Z, Yuan H, Yin Y, Zeng X, Bai R, Glazer RI (2006) 3-Phosphoinositide-dependent protein kinase-1 (PDK1) promotes invasion and activation of matrix metalloproteinases. BMC Cancer 6: 77 16551362 10.1186/1471-2407-6-77 PMC1459872

[bib68] Xie Z, Zeng X, Waldman T, Glazer RI (2003) Transformation of mammary epithelial cells by 3-phosphoinositide-dependent protein kinase-1 activates beta-catenin and c-Myc, and down-regulates caveolin-1. Cancer Res 63: 5370–5375 14500370

[bib69] Yin Y, Russell RG, Dettin LE, Bai R, Wei ZL, Kozikowski AP, Kopelovich L, Glazer RI (2005) Peroxisome proliferator-activated receptor delta and gamma agonists differentially alter tumor differentiation and progression during mammary carcinogenesis. Cancer Res 65: 3950–3957 15867396 10.1158/0008-5472.CAN-04-3990

[bib70] Zeng X, Xu H, Glazer RI (2002) Transformation of mammary epithelial cells by 3-phosphoinositide-dependent protein kinase-1 (PDK1) is associated with the induction of protein kinase Calpha. Cancer Res 62: 3538–3543 12068001

[bib71] Zhang GY, Ahmed N, Riley C, Oliva K, Barker G, Quinn MA, Rice GE (2005) Enhanced expression of peroxisome proliferator-activated receptor gamma in epithelial ovarian carcinoma. Br J Cancer 92: 113–119 15583697 10.1038/sj.bjc.6602244 PMC2361744

